# CD11c-mediated deletion of Flip promotes autoreactivity and inflammatory arthritis

**DOI:** 10.1038/ncomms8086

**Published:** 2015-05-12

**Authors:** Qi-Quan Huang, Harris Perlman, Robert Birkett, Renee Doyle, Deyu Fang, G. Kenneth Haines, William Robinson, Syamal Datta, Zan Huang, Quan-Zhen Li, Hyewon Phee, Richard M. Pope

**Affiliations:** 1 Division of Rheumatology, Department of Medicine, Northwestern University Feinberg School of Medicine, Chicago, Illinois 60611, USA; 2 Department of Pathology, Northwestern University Feinberg School of Medicine, Chicago, Illinois 60611, USA; 3 Department of Pathology, Mount Sinai Hospital School of Medicine, New York city, New York 10029, USA; 4 Division of Immunology and Rheumatology, Department of Medicine, Stanford University School of Medicine, VA Health Care System, Palo Alto, California 94304, USA; 5 Department of Biochemistry, College of Life Sciences, Wuhan University, Wuhan, Hubei 430072, China; 6 Department of Immunology and Internal Medicine, University of Texas Southwestern Medical Center, Dallas, Texas 75390, USA; 7 Department of Microbiology-Immunology, Northwestern University Feinberg School of Medicine, Chicago, Illinois 60611, USA

## Abstract

Dendritic cells (DCs) are critical for immune homeostasis. To target DCs, we generated a mouse line with *Flip* deficiency in cells that express cre under the CD11c promoter (CD11c-Flip-KO). CD11c-Flip-KO mice spontaneously develop erosive, inflammatory arthritis, resembling rheumatoid arthritis, which is dramatically reduced when these mice are crossed with *Rag*^*−/*−^ mice. The CD8α^+^ DC subset is significantly reduced, along with alterations in NK cells and macrophages. Autoreactive CD4^+^ T cells and autoantibodies specific for joint tissue are present, and arthritis severity correlates with the number of autoreactive CD4^+^ T cells and plasmablasts in the joint-draining lymph nodes. Reduced T regulatory cells (Tregs) inversely correlate with arthritis severity, and the transfer of Tregs ameliorates arthritis. This KO line identifies a model that will permit in depth interrogation of the pathogenesis of rheumatoid arthritis, including the role of CD8α^+^ DCs and other cells of the immune system.

## Abstract (web summary)

Dendritic cells are critical for initiation of immune responses and for induction of tolerance. Here the authors show that deletion of survival factor c-flip in CD11c-expressing cells subset perturbs CD8a^+^ dendritic cell, NK and macrophage pools, and leads to development of autoimmune arthritis.

## Graphical abstract

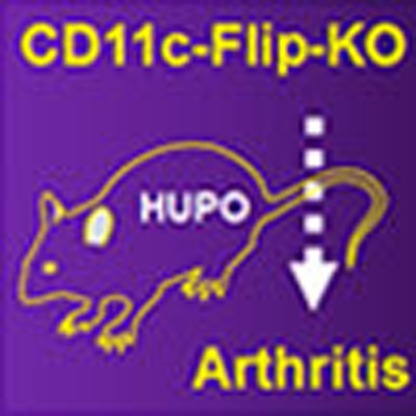

Dendritic cells (DCs) link the innate and adaptive immune systems. DCs, macrophages and B lymphocytes are capable of processing and presenting antigens to T lymphocytes; however, DCs are uniquely capable of priming naïve T cells. Classical or conventional DCs (cDCs) arising from haematopoietic precursors are identified by their high expression of CD11c (ref. 1). cDCs resident in lymphoid tissues including the thymus and spleen do not migrate, and they present foreign or self-antigens to T lymphocytes. Lymph nodes (LNs) also contain migratory DCs that migrate from the intestine, lung or skin when loaded with antigen^2^. The differentiation of DCs from common DC progenitor cells is transcriptionally regulated and dependent on cytokines, especially fms-related tyrosine kinase 3 ligand (Flt3L) and granulocyte–macrophage colony-stimulating factor (GM-CSF)^3^. Lymphoid tissue-resident DCs may be CD8α^+^ or CD8α^−^. The lymphoid tissue-resident CD8α^+^ subset cross-presents foreign or self-antigens loaded on major histocompatibility complex (MHC) class I complexes to CD8^+^ T cells, while CD8α^−^ DCs present peptides only in the context of MHC class II molecules^2^.

The role of DCs in the generation of tolerance is an area of active investigation. Thymic medullary cDCs may be involved in central tolerance, by virtue of cross-presentation of self-antigens from autoimmune regulator expressing (AIRE+) medullary thymic epithelial cells to T lymphocytes^4^,^5^,^6^. Supporting this mechanism, constitutive deletion of almost all cDCs employing a CD11c-Cre-mediated expression of diphtheria toxin A (ΔDC)^7^ resulted in impaired negative selection, although similar mice generated with CD11c-Cre BAC mice (cDC-less) did not exhibit such a defect^8^. cDCs in the thymus and periphery contribute to the development of T regulatory cells (Tregs), which are critical for peripheral tolerance^9^,^10^. A deficiency of Tregs results in fatal autoimmune disease in humans and mice^11^,^12^. The cDC-less, but not the ΔDC, mice demonstrated a reduction of Tregs in the spleen and mesenteric LNs^7^,^13^.

*Flip (CFLAR)*, identified as a rheumatoid arthritis (RA) risk allele^14^, is important in preventing death receptor-mediated apoptosis. To examine the *in vivo* role of *Flip* in DCs, mice were generated deleting *Flip* in CD11c^+^ cells (CD11c Flip knockout; CD11c-Flip-KO). On the basis of the development of autoimmunity in ΔDC mice^7^ and the potential role of Flip in resistance of DCs to death receptor-mediated cell death^15^,^16^, we anticipated that deletion of *Flip* in DCs would result in a marked reduction in DCs and the development of a lupus-like autoimmunity.

The CD11c-Flip-KO mice, however, developed a spontaneous, inflammatory, erosive arthritis. cDCs, particularly the CD8α^+^ subset, were reduced in the thymus, spleen and LNs before arthritis onset. The KO mice were lymphopenic, and CD4^+^ T cells from LNs draining the inflamed joints were autoreactive, and the mice developed autoantibodies to joint constituents. Splenic Tregs were reduced, and the number inversely correlated with arthritis severity, while adoptive transfer of Tregs ameliorated arthritis. Thus, the CD11c-Flip-KO line is a novel model that will permit the in-depth interrogation of the pathogenesis of RA.

## Results

### Deletion of Flip in CD11c cells

In order to determine the role of Flip in cDC, *Flip*^*flox/flox*^ mice were crossed with mice expressing GFP-Cre recombinase under the control of the CD11c promoter (*CD11*c^*cre*^)^17^, resulting in CD11c-Flip-KO mice. These mice were born in the expected Mendelian ratio. Body weight of the CD11c-Flip-KO mice was reduced by ∼15% at 4 weeks and 30% at ≥20 weeks. The mice demonstrated normal fertility, and no premature death was observed through 10 months of age. *Flip* deletion was determined using PCR employing purified splenocytes from *Flip*^*flox/+*^or *Flip*^*flox/flox*^ mice expressing *CD11*c^*cre*^. The partial deletion of the floxed *Flip* allele was clearly observed in both CD8α^+^ and CD8α^−^ cDCs, but minimally or not observed in the other cell types examined (Supplementary Fig. 1a).

### CD11c-Flip-KO mice develop spontaneous arthritis

**Figure 1:**
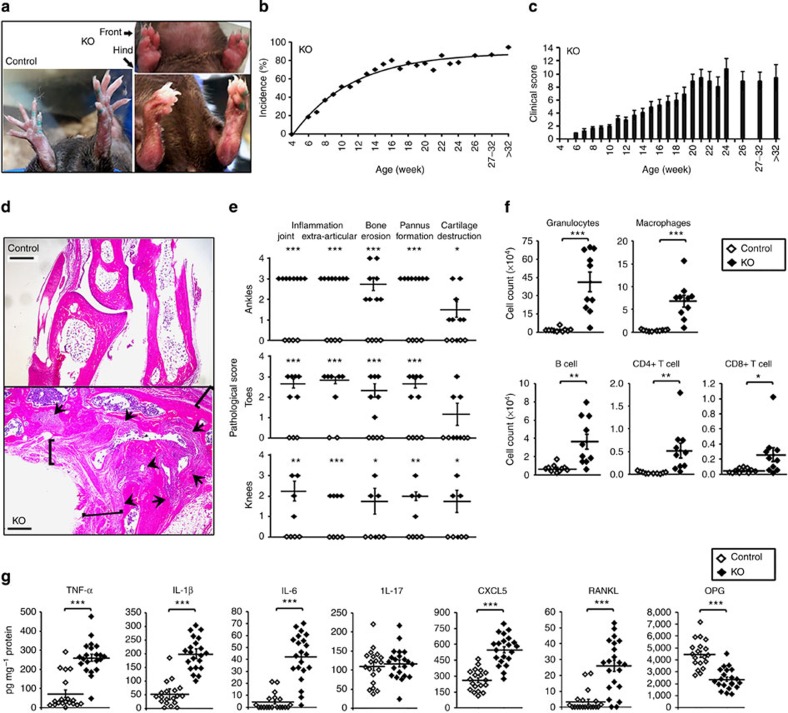
CD11c-Flip-KO mice develop spontaneous arthritis. (**a**) Representative joint swelling and flexion contraction in CD11c-Flip-KO (KO) mice. (**b**) Clinical incidence and (**c**) severity of spontaneous arthritis, *n*=16–57 at each time point. (**d**) Representative H&E histology of ankles of control and CD11c-Flip-KO (KO) mice. The arrowheads identify bone erosion and pannus formation, and the brackets indicate articular and extra-articular inflammation. Scale bar, 500 μm. (**e**) The comparison of histological scoring for ankles, toes and knees. (**f**) CD45^+^ inflammatory cells from ankles and knees were identified using flow cytometry employing antibodies to Ly6G, F4/80, CD19, CD4 and CD8. (**g**) Inflammatory mediators in ankle homogenates determined using ELISA. The concentration of each molecule was adjusted to total ankle homogenate protein (mg). For **c** through **g**, only the CD11c-Flip-KO mice with arthritis were included, which were compared with littermate controls. The values presented are the mean±1s.e. (*P<0.05, **P<0.01 and ***P<0.001, unpaired two-sided *t*-test).

Beginning at 6 weeks of age, the CD11c-Flip-KO mice spontaneously developed joint swelling, leading to peripheral joint deformities (Fig. 1a). Arthritis incidence and severity increased through 20 weeks (Fig. 1b,c), with no difference between males and females. The interphalangeal joints of the hind and front paws, ankles, wrists and knees were affected. Histologic examination revealed articular and extra-articular inflammation, and pannus, bone and cartilage destruction, which was not observed in the littermate controls (Fig. 1d,e). Using flow cytometry, granulocytes, macrophages, B lymphocytes and CD4^+^ and CD8^+^ T lymphocytes were increased in the joints of the CD11c-Flip-KO mice with arthritis compared with controls (Fig. 1f). Examination of the joint tissue from the mice demonstrated increased pro-inflammatory cytokines and chemokines in the KO mice; however, interleukin (IL)-17 was not increased and osteoprotegerin (OPG), which limits osteoclast activation, was reduced (Fig. 1g). Although they exhibited a modest increase in circulating neutrophils and monocytes (Supplementary Fig. 1b), by histologic examination there was no infiltration of neutrophils in the kidneys, liver, lung, thymus or small or large intestines.

### Reduction of cDCs in peripheral lymphoid organs

**Figure 2:**
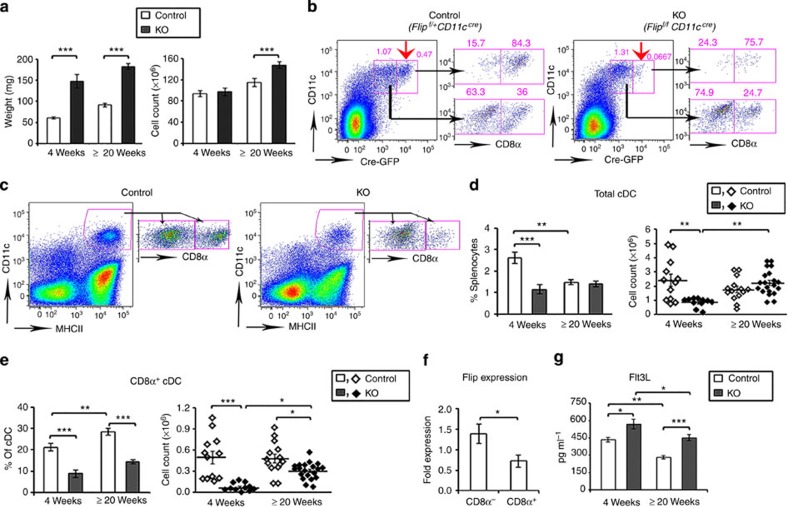
Decreased CD8α^+^ cDCs in spleens of CD11c-Flip-KO mice. (**a**) Increased spleen weight and cell number in CD11c-Flip-KO (KO) mice (*n*=26–36 per group). (**b**) Representative (of ≥3) flow cytometry gating for CD11c^+^Cre-GFP^hi^ cells. The red arrows identify the Cre-GFP^hi^ population. CD8α expression in both Cre-GFP^lo^ and Cre-GFP^hi^ population are indicated on the right for each panel. (**c**) Representative flow cytometry gating for total spleen cDCs and the CD8α^+^ subset at 4 weeks. The total cDCs are defined as CD64^−^CD11c^+^MHCII^+^ (**d**), and its CD8α^+^ subset (**e**), which was both analysed by percentage and cell numbers. (**f**) *Flip* expression determined using RT–PCR employing purified CD11c^+^MHCII^+^CD8α^+^ and CD8α^−^ cDCs (*n*=4). (**g**) The concentration of Flt3 ligand (Flt3L, pg ml^−1^) in the serum of mice was determined using ELISA (*n*=15 in each group, except for *n*=35 in the CD11c-Flip-KO (KO) mice ≥20 week). The values presented are the mean ±1 s.e. (**P<0.01 and ***P<0.001; (**a**,**b**,**f**) unpaired two-sided *t*-test; (**d**,**e**,**g**) ANOVA plus Tukey).

Studies were performed to understand the effect of *Flip* deletion in DCs on peripheral lymphoid organs. The spleen size was increased at 4 and ≥20 weeks in the KO mice (Fig. 2a), associated with an increase in CD64^+^F4/80^lo^CD11b^hi^ macrophages and Ly6G^+^ granulocytes, while the CD64^+^F4/80^hi^CD11b^lo^ red pulp macrophages were reduced at 4 weeks (Supplementary Fig. 2a,b). CD11c may also be expressed in NK cells, which were reduced at 4 and ≥20 weeks in the CD11c-Flip-KO mice (Supplementary Fig. 2c). The CD11c-driven Cre construct also expresses GFP. There was a clear deletion of GFP^hi^ cells in the CD11c^+^ population, which was enriched in CD8α^+^ cells, in the *Flip*^*f/f*^ mice compared with the *Flip*^*f/+*^ mice (Fig. 2b). Consistent with this observation, at 4 weeks the percentage and number of CD11c^+^MHCII^+^ cDCs were decreased, primarily because of a reduction (*P*<0.001, analysis of variance (ANOVA) plus Tukey) of the CD8α^+^ subset (Fig. 2c,d). By 20 weeks, while the total number of cDC was not different from the controls, the CD8α^+^ subset was still significantly (*P*<0.001, ANOVA plus Tukey) reduced (Fig. 2e). The CD8α^+^ subset may have been selectively affected because there is less *Flip* mRNA in these cells (Fig. 2f) and because Cre was more strongly expressed (Fig. 2b), likely resulting in more efficient *Flip* deletion. There was no difference in the percentage or number of plasmacytoid DCs at 4 weeks, although they were increased at 20 weeks (Supplementary Fig. 2,d). Similar but less dramatic changes of cDCs, macrophages and granulocytes were observed in the mixed lymph nodes (MxLNs), a combination of cervical, brachial, axillary and inguinal LNs, from the CD11c-Flip-KO mice (Supplementary Fig. 3a–f). Flt3L, critical for DC development in the periphery, was increased in the circulation of the CD11c-Flip-KO mice at 4 and ≥ 20 weeks (Fig. 2g).

### Flip is necessary for DC development

**Figure 3:**
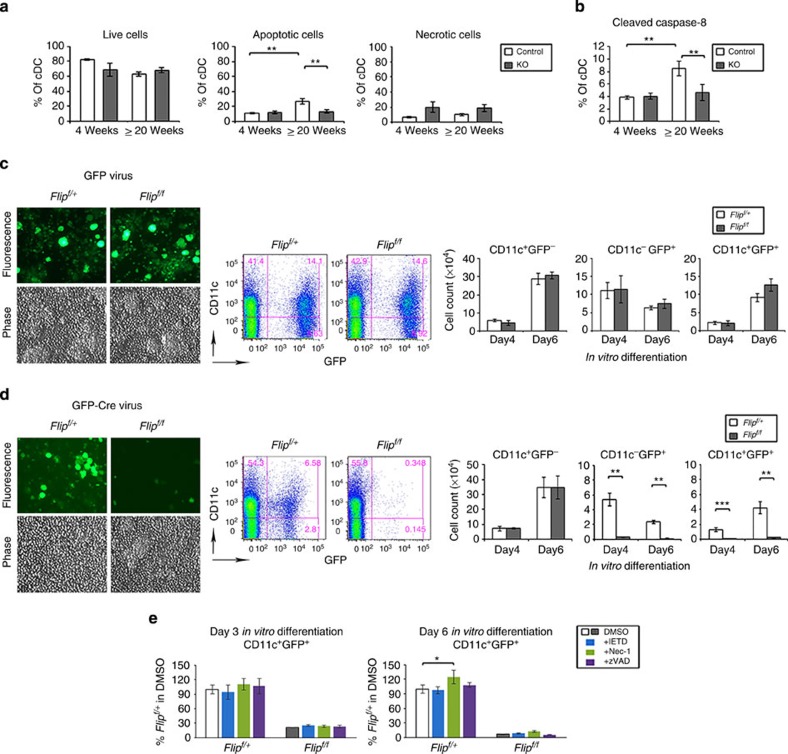
Flip is necessary for DC development. (**a**) cDCs *in vivo* apoptosis and necrosis in spleen were examined with 7AAD and Annexin V, gating on the CD64^−^CD11c^+^MHCII^+^ DC population in the CD11c-Flip-KO (KO) and littermate control mice (*n*=4–5 per group). Live cells were identified as 7AAD^−^Annexin V^−^, apoptotic cells were 7AAD^−^Annexin V^+^ and necrotic cells are 7AAD^+^. (**b**) The caspase-8 activation in cDCs was determined employing an antibody to cleaved caspase-8, (*n*=5–6 per group). (**c**–**e**) Lineage-negative (Lin^−^) haematopoietic stem cells from the bone marrow of *Flip*^*f/+*^ *or Flip*^*f/f*^ mice were isolated and infected with recombinant retroviral vectors expressing GFP alone (**c**) or GFP-Cre (**d**) followed by *in vitro* differentiation in a medium containing Flt3L and GM-CSF. Representative fluorescence microscopy and flow histograms (day 6 post differentiation) for each viral infection is presented. The numbers of CD11c^+^GFP^−^, CD11c^−^GFP^+^ or CD11c^+^GFP^+^ cells were determined using flow cytometry. (**e**) Lin^−^ haematopoietic stem cells from the bone marrow of *Flip*^*f/+*^ *or Flip*^*f/f*^ mice (*n*=3 per group) were infected with the GFP-Cre retrovirus and differentiated as in **d**, except differentiation was performed in the dimethylsulphoxide (DMSO) control medium or a medium containing optimal concentrations of IETD.fmk, necrostatin-1 or zVAD.fmk. Cells were harvested at 3 or 6 days of differentiation. The data were analysed adjusting the values for the Flip^f/+^ cells in control medium to 100%. ( (**c**–**e**) *n*=3 per group from two independent experiments). (*P<0.05, **P<0.01 and ***P<0.001; for **a**,**b**,**e**, ANOVA plus Tukey; for **d**, unpaired two-sided *t*-test).

Since Flip was increased in DCs resistant to Fas-mediated apoptosis^15^,^16^, spleens of CD11c-Flip-KO mice were examined to identify apoptosis in cDCs at 4 and ≥20 weeks. There was no difference in the percent live, apoptotic, active caspase 8-positive or necrotic cDCs between the CD11c-Flip-KO and control mice at 4 weeks (Fig. 3a). At ≥20 weeks there was an increase in apoptotic and active caspase 8-positive cDCs in the controls, compared with the KO mice (Fig. 3b). Therefore, increased apoptosis does not appear to be the cause for the reduction in DCs in the KO mice.

Since the genotyping demonstrates only a partial reduction of *Flip in vivo* (Supplementary Fig. 1a), and the half-life of splenic DCs is <3 days^18^, the role of Flip in the differentiation of DCs was examined. Lineage-negative haematopoietic stem cells were isolated from *Flip*^*f/+*^ or *Flip*^*f/f*^ mice as previously described by us^19^, and infected with recombinant retroviral vectors expressing green fluorescent protein (GFP) alone or GFP plus Cre and then differentiated into DCs. Using fluorescence microscopy on day 6 following differentiation, CD11c^+^GFP^+^ cells were comparable employing cells from Flip^*f/+*^ or Flip^*f/f*^ mice (Fig. 3c). Using flow cytometry on days 4 and 6 there was no difference in the numbers of CD11c^+^GFP^−^, CD11c^−^GFP^+^ or CD11c^+^GFP^+^ cells between the cells from *Flip*^*f/+*^ or *Flip*^*f/f*^ mice. In contrast, following infection with the GFP-Cre-expressing retroviral vector, there was a significant (*P*<0.01) reduction of both CD11c^−^GFP^+^ and CD11c^+^GFP^+^ cells in cultures from the *Flip*^*f/f*^ compared with *Flip*^*f/+*^ mice (Fig. 3d). There was no difference of the CD11c^+^GFP^−^ cells between the two groups. There was no increase inCD11c^+^GFP^+^ DCs, when the differentiation was performed in the presence of optimal concentrations of IETD.fmk, zVAD.fmk or necrostatin-1, although there was a slight increase in viability of the control cells with necrostatin-1 (Fig. 3e). These observations demonstrate that Flip is required for DC differentiation, and that survival was not rescued by caspase or RIP1 inhibition.

### CD4^+^ T cells from CD11c-Flip-KO mice are autoreactive

To identify the mechanisms that may permit the spontaneous arthritis, lymphocytes were assessed at 4 weeks and contrasted with the results at ≥20 weeks (Table 1). Both percentage and total numbers of CD4^+^ and CD8^+^ lymphocytes were reduced in the spleens and MxLNs, with the exception that the total number of CD4^+^ lymphocytes was increased in the MxLNs at ≥20 weeks of age. There was a modest increase in Th17 lymphocytes and reduction in Th1 lymphocytes at ≥20 weeks in the spleens of the KO mice.

**Figure 4:**
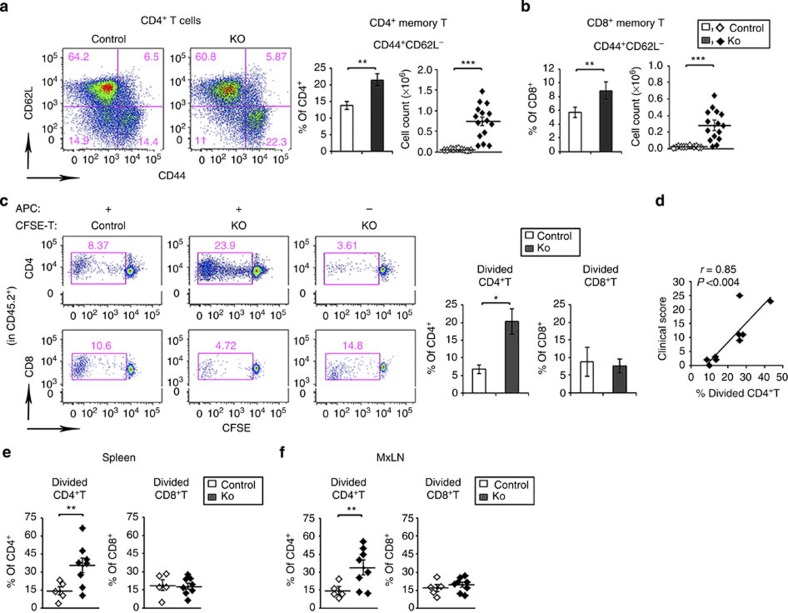
CD4^+^ T cells from CD11c-Flip-KO mice are autoreactive. (**a**,**b**) pLNs were examined for memory T cells (CD44^+^CD62L^−^) in CD4^+^ (**a**) and CD8^+^ (**b**) T-cell populations at ≥20 weeks in the CD11c-Flip-KO (KO) and control mice. (**c**) *In vitro* syngeneic mixed lymphocyte response was employed to identify autoreactive T cells. T cells from the pLNs and brachial LNs of ≥20 week CD11c-Flip-KO mice were CFSE-labelled and represent the responder T cells (3 × 10^5^). The antigen-presenting cells (APCs) are T-cell-depleted spleen cells from CD45.1 mice (3 × 10^5^). After co-culture for 5–7 days, cell division was determined by the dilution of CFSE. The panels on the left are representative flow gating defining the divided cells present in the CD45.2^+^ CD4^+^ or CD8^+^ T-cell populations. The panels on the right are the analysis of KO (*n*=9) and control mice (*n*=4). (**d**) Correlation (Pearson’s) between the severity of arthritis in Flip-DC-KO mice just before being killed and the percent of divided CD4^+^ T cells as defined in **c**. Identification of autoreactive T cells by adoptive transfer of CFSE-labelled T cells from the (**e**) spleen and (**f**) MxLNs of CD11c-Flip-KO or littermate control mice (≥20 weeks, CD45.2^+^) into CD45.1 recipients. The autoreactive T cells were defined using flow cytometry as defined in **c**. The spleens and MxLNs from the recipients were examined 8–11 days after transfer. Each data point represents an individual recipient mouse that received T cells from a single donor (*P<0.05, **P<0.01 and ***P<0.001, unpaired two-sided *t*-test).

Popliteal LNs (pLNs) draining the inflamed ankles and hind paws were examined for memory T cells. CD44^+^CD62L^−^ CD4^+^ and CD8^+^ T cells were increased in the pLNs at ≥20 weeks (Fig. 4a,b). Therefore, the presence of autoreactive T cells in the CD11c-Flip-KO mice or controls was determined, employing the syngeneic mixed lymphocyte response. T cells from pLNs and brachial LNs from mice ≥20 weeks were labelled with carboxyfluorescein diacetate succinimidyl ester (CFSE) and co-cultured using T-cell-depleted spleen cells from syngeneic CD45.1 mice as the antigen-presenting cells. On days 5–7, increased proliferation of CD4^+^, but not CD8^+^, T cells from the CD11c-Flip-KO was observed (*P*<0.05, unpaired two-sided *t*-test, Fig. 4c). The percentage of proliferating CD4^+^ T cells correlated with the severity of arthritis, defined by the clinical score (*P*<0.004, Pearson’s correlation, Fig. 4d). Similar experiments were performed adoptively transferring CFSE-labelled T cells into syngeneic CD45.1 mice. When spleens or MxLNs of the recipients were harvested, increased proliferation of the CD11c-Flip-KO CD4^+^, but not CD8^+^, lymphocytes was observed (Fig. 4e). Homeostatic proliferation of adoptively transferred T cells into *Rag*^*−/−*^ mice demonstrated no difference between the KO and control lymphocytes (Supplementary Fig. 4a). Furthermore, there was no difference in anti-CD3/-CD28-induced proliferation of T cells between CD11c-Flip-KO and control mice (Supplementary Fig. 4b). These observations suggest the presence of autoreactive CD4^+^ lymphocytes in the CD11c-Flip-KO mice.

### No defect of antigen presentation by CD11c^+^ DCs from KO mice

Since CD4^+^ T cells were autoreactive, the functional consequence of the reduction of *Flip* in CD11c^+^ DCs on antigen presentation was examined employing CFSE-labelled CD4^+^OTII^+^ T cells, which are specific for ovalbumin (OVA) peptide 323–339 in the context of I-A^b^. Flt3L-differentiated bone marrow-derived CD11c^+^ DCs exhibited a partial deletion of *Flip* (Supplementary Fig. 5a), similar to DCs isolated from the spleen. There was no significant difference in the ability of these cells derived from KO mice to present antigen to OTII^+^ T cells compared with the control DCs, in the presence or absence of lipopolysaccharide (Supplementary Fig. 5b). Furthermore, CD11c^+^ DCs isolated from spleens exhibited no significant difference between CD11c-Flip-KO and control mice in their ability to activate OTII^+^ T cells, when the DCs were isolated before or after the onset of arthritis (Supplementary Fig. 5c,d). These observations suggest that a difference in the ability of the residual CD11c^+^ DCs in the KO mice to present antigen to CD4^+^ T cells was not the cause of arthritis.

### Alterations in the thymus precede development of arthritis

**Figure 5:**
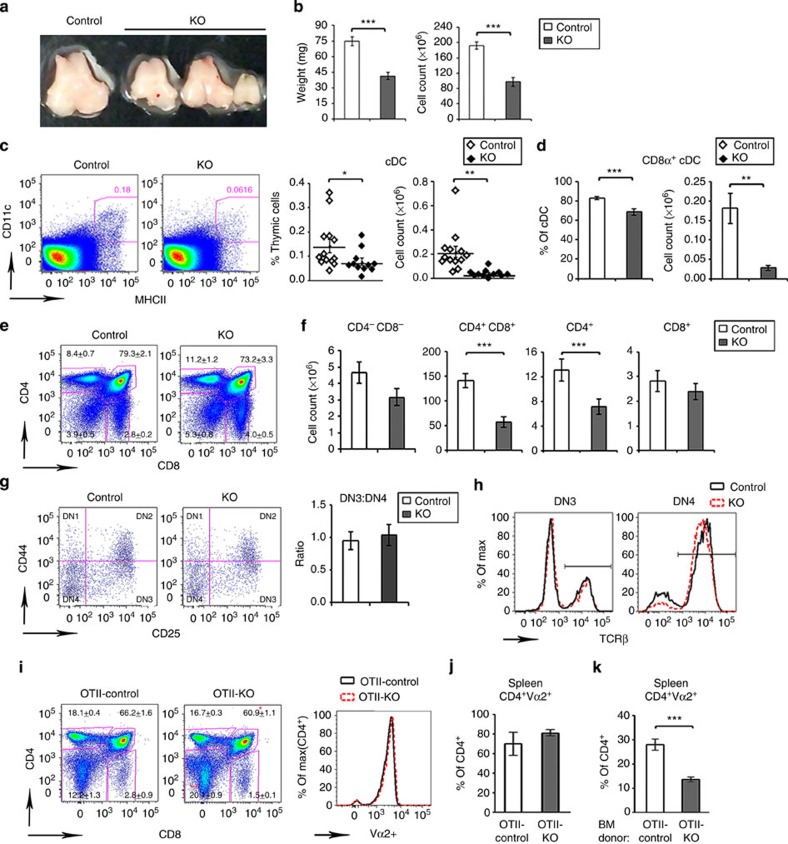
Alterations in the thymus precede development of arthritis in the CD11c-Flip-KO mice. (**a**,**b**) Representative photos of thymi, which were analysed by weight and cell count (*n*=25–27 per group). (**c**) Identification of total cDC and (**d**) the CD8α^+^ subset, presented as percent and total number. cDCs were defined as CD64^−^CD11c^+^MHCII^+^ (*n*=11–13 per group). (**e**,**f**) Thymocytes were characterized for the (**e**) percent and (**f**) number of CD4 and CD8 as CD4^−^CD8−(DN), CD4^+^CD8^+^ (DP) and CD4 or CD8 SP (*n*=8–9 per group). The numbers in the histogram (**e**) represent the means±s.e for each population. (**g**) Development from the DN1 through DN4 of CD4^−^CD8^−^ thymocytes determined by antibodies to CD25 and CD44, and the ratio of DN3/DN4 (*n*=8–9 per group). (**h**) Representative overlay of intracellular TCR-β expression in DN3 and DN4 cells (*n*=3 per group). (**i**) Comparison of the thymocytes in mice crossed with OTII, genotyped as OTII-*Flip*^*flox/flox*^, *CD11c*^*+*^ (OTII-control) and OTII-*Flip*^*flox/flox*^, *CD11c*^*cre*^ (OTII-KO; *n*=4 per group). The expression of OTII transgene Vα2 in CD4^+^ T cell is overlaid (right panel). (**j**) Assessment of clonotypic CD4^+^Vα2^+^ T cells in spleens of OTII-control mice and OTII-KO mice (*n*=4 per group) and (**k**) of irradiated RIP-mOVA recipients that received the BM from OTII-control or OTII-KO donors (*n*=7–10 per group). The data are presented as percent of CD4^+^Vα2^+^ cells of all CD4^+^ cells. (*P<0.05, **P<0.01 and ***P<0.001, unpaired two-sided *t*-test).

Because of the T-cell abnormalities in the periphery, the thymi of KO and control mice were examined at 4 weeks. The thymi of the KO mice were small (Fig. 5a), with significant (*P*<0.001, unpaired two-sided *t*-test) reduction of both weight and cell number (Fig. 5b). While histologic examination did not reveal differences, other than size, in the appearance of the cortex or medulla, the percent and total number of CD11c^+^MHCII^+^ cDCs were decreased (*P*<0.05–0.01, unpaired two-sided *t*-test) in KO mice (Fig. 5c), as was the percent and number of CD8α^+^ cDCs (*P*<0.01-0-001, unpaired two-sided *t*-test, Fig. 5d).

Since thymic DCs may contribute to central tolerance^4^,^20^, the development of thymocytes was examined. There was no difference in the percentage of CD4, CD8-double-negative (DN) or CD4 or CD8-single-positive (SP) thymocytes between the KO and control mice (Fig. 5e). However, the numbers of double-positive (DP) and CD4 and CD8 SP thymocytes were reduced in CD11c-Flip-KO mice (Fig. 5f). Transition from the DN1 to the DN4 stage was normal (Fig. 5g). Expression of intracellular T-cell antigen receptor (TCR)-β chain was similar at DN3 and DN4 stages, suggesting that development at the checkpoint mediated by the pre-TCR is normal (Fig. 5h). To eliminate compensatory changes in the TCR repertoire, we introduced the OTII transgene into the CD11c-Flip-KO mice. A modest decrease in the percentage of DP and an increase in DN cells was observed, but percentage of CD4 SP thymocytes was similar between OTII-control and OTII-CD11c-Flip-KO mice (Fig. 5i), suggesting that the generation of CD4 SP thymocytes via positive selection was not impaired. Bone marrow chimeras using RIP-mOVA mice as hosts and OTII-control or OTII-CD11c-Flip-KO bone marrows as donors were generated. Examination of splenocytes from OTII-KO donors (Fig. 5j) and the RIP-mOVA recipients (Fig. 5k) did not reveal an increased percentage of the clonotype Vα2^+^CD4^+^ OTII T cells in the RIP-mOVA recipients, suggesting that negative selection was not impaired in the CD11c-Flip-KO mice.

Since no defects in thymic selection were identified, early thymic progenitors (ETPs, Kit^+^CD25^−^CD4^−^CD8^−^) were examined in the thymus. The number (*P*<0.05, unpaired two-sided *t*-test) of ETPs was reduced at 4 weeks of age (Supplementary Fig. 6a). This decrease in ETPs was not due to a reduction in bone marrow haematopoietic stem cells, defined as Lin^−^Kit^+^Sca1^+^Flt3^−^ (LSK) that were significantly increased (*P*<0.001, unpaired two-sided *t*-test) in the CD11c-Flip-KO mice. However, the percent that differentiated into Lin^−^Kit^+^Sca^+^Flt3^hi^ (LMPP), capable of emigrating from the bone marrow to the thymus, was reduced in the CD11c-Flip-KO mice (Supplementary Fig. 6b,c). These observations do not identify a reduction of thymic progenitors in the bone marrow, but suggest that there may be a block in the maturation from LSK to LMPP.

### Autoantibodies to joint components in CD11c-Flip-KO mice

**Figure 6:**
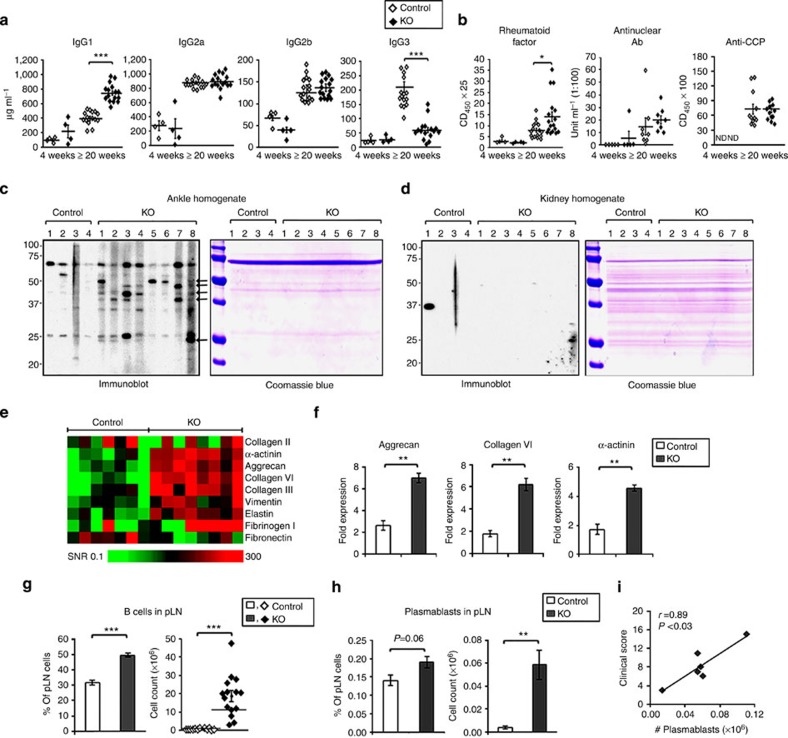
Autoantibodies and increased B cells and plasmablasts in CD11c-Flip-KO mice. (**a**) Quantification of serum IgG subclasses by ELISA (μg ml^−1^) in the CD11c-Flip-KO (KO) and control mice. (**b**) ELISA quantification of serum RF, ANA and antiCCP (lower panel). Values are presented as OD_450_ × serum dilution for RF and anti-CCP and units for ANA. (**c**,**d**) Immunoblotting of ankle (**c**) and kidney (**d**) homogenates from *Rag*^*−/−*^ mice employing individual serum randomly selected from control and CD11c-Flip-KO mice (≥20 weeks) and a duplicated tissue blot stained with Coomassie blue R250. The arrows identify protein bands common between individual CD11c-Flip-KO but not in control mice. The blots are representative of three independent experiments. (**e**,**f**) Antibodies identified by autoantigen array. (**e**). Heatmap of IgG antibodies to joint-related antigens, generated as the signal-to-noise ratio (SNR) for each antigen (controls *n*=7, CD11c-Flip-KO mice ≥20 weeks with arthritis, *n*=8). (**f**) Three autoantibodies in the array were significantly increased following the Bonferroni correction in the CD11c-Flip-KO mice. (**g**,**h**) pLNs were examined for B cells (*n*=12–15 per group) and plasmablasts (*n*=6 per group) from mice ≥20 weeks. B cells were defined as CD19^+^B220^+^ and plasmablasts as CD19^+^B220^+^CD138^+^ in the CD64^−^CD11b^−^ population. (**i**) Correlation (Pearson’s) between the number of pLN plasmablasts and the arthritis clinical score for CD11c-Flip-KO mice just before killing. The values represent the mean±1 s.e. (*P<0.05, **P<0.01 and ***P<0.001, all panels unpaired two-sided *t*-test and in **f** unpaired two-sided *t*-test followed by the Bonferroni correction).

Since autoreactive T cells were identified, serum antibody levels were examined at 4 and at ≥20 weeks. IgG1 was significantly (*P*<0.001, unpaired two-sided *t*-test) increased and IgG3 reduced (*P*<0.001, unpaired two-sided *t*-test) at ≥20 weeks, while IgG2b and IgG2c were not different at either time point (Fig. 6a). The mice were examined for autoantibodies detected in RA. At ≥20 weeks of age, rheumatoid factor (RF) was significantly (*P*<0.05, unpaired two-sided *t*-test) increased, although the values in only three mice (17%) were elevated (Fig. 6b). There was no increase in antinuclear antibodies (ANAs). Since antibodies to a panel of citrullinated peptides from proteins that may contribute to the pathogenesis of RA, including fibrinogen, histones, enolase and vimentin, were detected in the KO mice, antibodies to cyclic citrullinated peptide (CCP) (Fig. 6b) were screened for, but none were identified.

Since the CD11c-Flip-KO mice uniquely develop inflammatory arthritis, we determined whether circulating autoantibodies to joint tissue were present. Joint tissue was isolated from the ankles of *Rag*^*−/−*^ mice, subjected to SDS–PAGE and probed with the sera from individual mice. Antibodies to joint tissues, but not to the kidney or liver, were present in sera from the KO mice ≥20 weeks of age, but not in the controls (Fig. 6c,d and Supplementary Fig. 7 for full-size scans of immunoblots). Therefore, a multiplex assay employing 123 proteins, including a number selectively expressed in joint tissue was employed. Increased antibodies to a number of proteins expressed in the cartilage, bone and muscle were identified (Fig. 6e). Following the Bonferroni correction (*p*_c_) only antibodies to aggrecan (*p*_c_<0.002), collagen IV (*p*_c_<0.004) and α-actinin (*p*_c_<0.002) were significantly increased in the sera of CD11c-Flip-KO mice (Fig. 6f). Consistent with the presence of autoantibodies, B lymphocytes were increased in the MxLNs at 4 and ≥20 weeks (*P*<0.05–0.001, unpaired two-sided *t*-test), and the spleen and pLNs (*P*<0.001, unpaired two-sided *t*-test) at 20 weeks (Table 1 and Fig. 6g). Plasmablasts were increased in the pLNs of the KO mice, and the number was highly correlated (*r*=0.89, *P*<0.001, Pearson correlation) with the clinical arthritis score at ≥20 weeks (Fig. 6h,i). These data support the role of B cells in this model.

### T and B lymphocytes are necessary for arthritis progression

**Figure 7:**
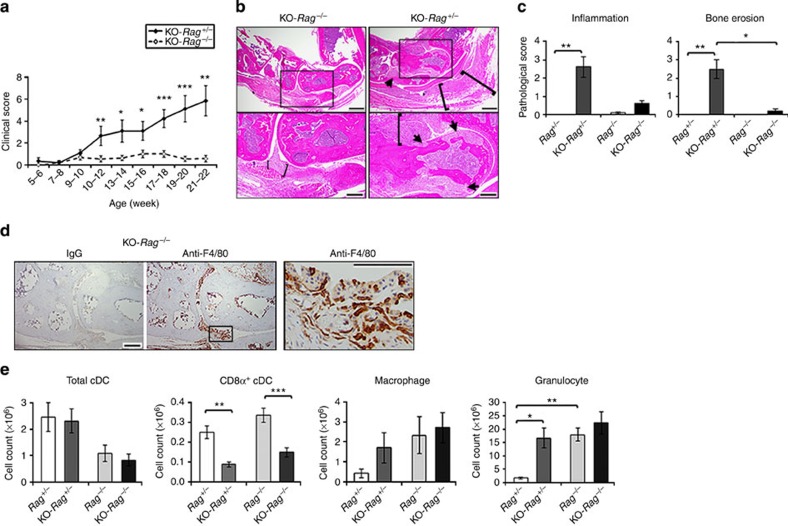
Reduced arthritis progression in KO-*Rag*^*−/−*^ mice. The CD11c-Flip-KO mice were crossed with *Rag*^*−/−*^ mice to generate the KO-*Rag*^*−/−*^ line. (**a**) The spontaneous development of arthritis in KO-*Rag*^*−/−*^mice was compared with the littermate KO-*Rag*^*+/−*^ mice (*n*=7–10 KO-*Rag*^*+/−*^ mice and 7–16 KO-*Rag*^*−/−*^ at the observed time points). No arthritis was observed in the *Rag*^*−/−*^*or Rag*^*+/−*^ mice. (**b**) Representative (of four to five per group) H&E histology of ankles of KO-*Rag*^*−/−*^ mice and KO-*Rag*^*+/−*^ mice. The arrowheads identify bone erosion and pannus formation, and the brackets indicate inflammation. The boxed areas in upper panels (scale bar, 500 μm) are enlarged in the lower panels (scale bar, 200 μm). (**c**) The analysis of inflammation and bone erosion scores for ankles harvested from *Rag*^*−/−*^, KO-*Rag*^*−/−*^ and KO-*Rag*^*+/−*^ mice (*n*=4–5 mice for each group) harvested at the termination of the experiment (21–22 weeks). (**d**) Representative (of *n*=5) immunohistochemistry of joints from CD11c-Flip-KO mice for F4/80^+^ macrophages (scale bar, 200 μm). The boxed area in F4/80 staining is enlarged in the right panel (scale bar, 100 μm). (**e**) At the time of killing, spleens examined for DCs, macrophages and granulocytes (*n*=4–5 per group). The values presented are mean ±1 s.e. (*P<0.05, **P<0.01 and ***P<0.001; (**a**) unpaired two-sided *t*-test; (**c**) ANOVA plus Dunn and (**e**) ANOVA plus Tukey).

The presence of autoreactive T cells and autoantibodies suggests a role for T and B lymphocytes in the development of arthritis in the CD11c-Flip-KO mice. To examine this possibility directly, CD11c-Flip-KO mice were crossed with *Rag*^*−/−*^ mice (KO-*Rag*^*−/−*^ mice), which lack both T and B lymphocytes. Although up to 55% of the KO-*Rag*^*−/−*^ mice developed arthritis, in contrast to the CD11c-Flip-KO mice, the arthritis in the KO-*Rag*^*−/−*^ mice was significantly (*P*<0.05–0.001, unpaired two-sided *t*-test) less severe, and in some mice the arthritis spontaneously improved or resolved by 21–22 weeks (Fig. 7a). When examined histologically, the KO-*Rag*^*−/−*^ mice exhibited less inflammation and developed almost no bone erosions (Fig. 7b,c). Macrophages, but not neutrophils, infiltrated the joints of the CD11c-Flip-KO mice (Fig. 7d). Examination of the spleens confirmed that the CD8α^+^ cDC subset was significantly reduced in the KO-*Rag*^*−/−*^ and KO-*Rag*^*+/−*^ mice compared with *Rag*^*−/−*^ and *Rag*^*+/−*^ mice (Fig. 7e). Granulocytes and macrophages were increased in the spleens of the KO-*Rag*^*−/−*^, KO-*Rag*^*+/−*^ and the *Rag*^*−/−*^ mice comparably (Fig. 7e).

### Reduced Tregs permit arthritis development

**Figure 8:**
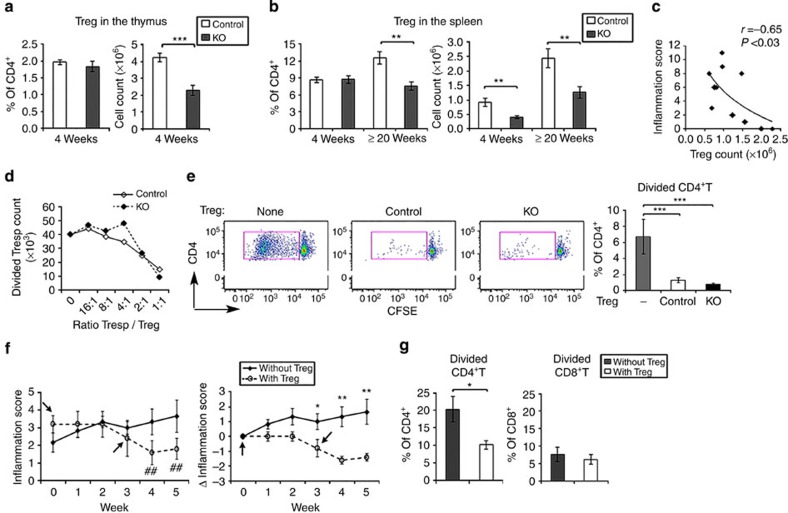
Reduced Tregs in CD11c-Flip-KO mice contribute to the pathogenesis of arthritis. (**a**) The percent and number of Tregs in the thymus (*n*=15 per group), defined as CD4^+^CD25^+^Foxp3^+^ in the CD11c-Flip-KO (KO) and control mice. (**b**) Tregs in spleens at 4 weeks (*n*=11) and ≥20 weeks (*n*=6–9), defined as CD4^+^CD25^+^Foxp3^+^. (**c**) Correlation (Pearson’s) between the number of spleen Tregs and the inflammation score of arthritis in CD11c-Flip-KO mice (≥20 week) determined just before killing. (**d**) The *in vitro* suppressive function of Tregs. The CD45.2^+^CD4^+^CD25^+^ T cells from CD11c-Flip-KO or control mice were co-cultured with CFSE-labelled CD45.1^+^CD4^+^D25^−^ Tresp cells at indicated ratios on anti-CD3 mAb-coated plates for 3 days, and examined for the CFSE dilution of CD4^+^ T cells. The results are representative of three experiments. Cell number was calculated by flow counting beads. (**e**) The *in vitro* suppressor function by Tregs for autoreactive CD4^+^T-cell proliferation, determined by *in vitro* syngeneic mixed lymphocyte response same as in Fig. 4. Results from three independent experiments, *n*=3 without Tregs and *n*=5 with Tregs. (**f**)The *in vivo* Treg function. CD11c-Flip-KO mice (*n*=5) were adoptively transferred with two dosages (∼ 2 × 10^6^ per dose) of CD4^+^CD25^+^ T cells isolated from the spleens of CD45.1 mice at 0 and 3 weeks. Six age-matched CD11c-Flip-KO mice served as the no-treatment controls. The values in the left panel represent the mean ±1 s.e of the inflammation scores, ^##^*P*<0.01 (paired *t* test) compared with the score at week 0. The data in right panel reflect the change in the inflammation score (Δ). (**g**) *In vitro* syngeneic mixed lymphocyte response. The T cells from the pLNs and brachial LNs of the Treg-treated mice (in **f**) were CFSE-labelled and incubated with syngeneic APCs to identify autoreactive T cells. The values for the autoreactive T cells of CD11c-Flip-KO mice without Treg treatment are same as presented in Fig. 4c, which are used here for comparison (*P<0.05, **P<0.01 and ***P<0.001, unpaired two-sided *t*-test, except(**e**) ANOVA plus Tukey).

The presence of Tregs was examined further since cDC may contribute to their development and because they may suppress the development of autoreactive T cells. At 4 weeks of age the number, but not percent, of natural thymic Tregs was significantly (*P*<0.001, unpaired two-sided *t*-test) reduced in the CD11c-Flip-KO mice (Fig. 8a). In the spleens, the number of Tregs was also reduced at 4 weeks, while both number and percent were decreased at ≤20 weeks (*P*<0.01, unpaired two-sided *t*-test; Fig. 8b). Supporting their potential role in the pathogenesis of arthritis, at 20 weeks the number of spleen Tregs, but not total CD3^+^CD4^+^ T cells, inversely correlated with the inflammation score (*r*=−0.65, *P*<0.03, Pearson’s correlation, Fig. 8c). Nonetheless, the ability of isolated Tregs from the CD11c-Flip-KO mice to suppress T-cell proliferation (Fig. 8d) or autoreactive CD4^+^ T cells (Fig. 8e) was not reduced. These observations suggest that a defect in cDC-mediated Treg development contributed to the pathogenesis of the arthritis in the CD11c-Flip-KO mice.

Experiments were performed to determine the effect of adoptive transfer of Tregs into CD11c-Flip-KO mice with arthritis. The adoptive transfer of syngeneic Tregs from CD45.1^+^ controls resulted in a significant (*P*<0.05–0.01, two-sided *t*-test) reduction in inflammation, compared with untreated, age-matched CD11c-Flip-KO mice, that was first noted 3 weeks after transfer, with further improvement at 4 and 5 weeks (Fig. 8f). There was a significant reduction in autoreactive CD4^+^, but not CD8^+^, T cells in the LNs draining the inflamed joints of the mice treated with Tregs, compared with CD11c-Flip-KO mice that were not treated (Fig. 8g) These observations demonstrate a critical role for reduced Tregs in the pathogenesis of arthritis observed in the CD11c-Flip-KO mice.

## Discussion

The Flip-DC-KO mice developed inflammatory, erosive polyarthritis. The deletion of *Flip* in DCs selectively reduced the CD8α^+^ DC subset in lymphoid tissues. The CD11c-Flip-KO mice were mildly lymphopenic, which may permit homeostatic proliferation^21^, identified as increased peripheral memory T cells, which included potentially autoreactive T cells that escaped the thymus. Data from the KO-*Rag*^*−/−*^mice suggest that in the CD11c-Flip-KO mice macrophages may initiate a mild self-limited arthritis, which may result in the release of antigens from the cartilage and bone. In the CD11c-Flip-KO mice, these joint-associated antigens may activate autoreactive CD4^+^ T cells that escaped negative selection, promoting the production of autoantibodies directed against antigens within the joint, resulting in a progressive, destructive arthritis. Supporting this interpretation, arthritis was markedly reduced in the KO-*Rag*^*−/−*^ mice. The reduction of Tregs was at least permissive, since adoptive transfer of Tregs from control mice suppressed arthritis and T-cell autoreactivity.

The mechanism responsible for the reduction of DCs, particularly the CD8α^+^ subset, in the KO mice may be multifactorial. Flip is increased in DCs resistant to Fas-mediated cell death^15^,^16^, and DCs are sensitive to FasL-mediated apoptosis^17^. Increased DC apoptosis in the spleens of CD11c-Flip-KO mice cannot be excluded since apoptotic cells may be rapidly cleared. Both CD8α^+^ and CD8α^−^ splenic DCs demonstrated only partial deletions of *Flip*, suggesting that the cells that were identified *in vivo* may still express Flip. Furthermore, since DC half-life *in vivo* is short, 1.5 days for CD8α^+^ DCs and between 2 and 2.9 days for CD8α^−^ DCs^18^, it may be difficult to achieve complete deletion of Flip before cell turnover. Unexpectedly, our data demonstrate that Flip is necessary for the differentiation of DCs, and the lack of DC differentiation was not protected by caspase or RIP1 inhibition. It is possible that lack of *Flip* prevents progression from macrophage DC progenitors or common DC progenitors^22^. In summary, our data support a block of DC differentiation, rather than increased apoptosis in the CD11c-Flip-KO mice. The increase of cDCs in the CD11c-Flip-KO mice between 4 and ≥20 weeks is likely due to the marked inflammatory response, which may contribute to reduced apoptosis, compared with the controls, and the increased circulating Flt3L, which is necessary for DC homeostasis^22^.

Prior studies have demonstrated that alterations of cDCs promote autoimmunity. Manipulations that increase DC survival or function consistently promote systemic autoimmunity, in some cases resembling lupus-like disease with nephritis and positive ANAs and in others ankylosing spondylitis with colitis^17^,^23^,^24^,^25^,^26^. Deletion of essentially all cDCs in the ΔDC and the cDC-less mice resulted in increased IgG1 (refs 7, 13), and reduced IgG3 (ref. 7), similar to the CD11c-Flip-KO mice. The ΔDC mice demonstrated positive ANA infiltration of intestines and kidney with CD4^+^ T cells^7^, which were not observed in the CD11c-Flip-KO mice. Although other immunologic abnormalities were identified, *Batf3*−/−, *Irf8*−/− and CD205-DTR mice, each demonstrated reduction of CD8α^+^ DCs; however, neither autoimmunity nor lymphopenia were documented^27^,^28^,^29^. In contrast, mice expressing a DC-selective DN GRPase Rac1 exhibited both a defect in the uptake and subsequent cross-presentation of apoptotic cellular antigens and a reduction in CD8α^+^ DCs, which permitted the accumulation of autoreactive CD8^+^ T cells, but not spontaneous autoimmune disease^30^.

Several lines of evidence support the role of B lymphocytes in the pathogenesis of the arthritis. IgG1, which is T-cell-dependent, was elevated, and B lymphocytes were increased in the MxLNs, pLNs, spleens and the inflamed joints. Further, plasmablasts were increased in the pLNs draining inflamed joints and were highly correlated with clinical severity. Although RF was detected in 17% of mice, no ANA or anti-CCP antibodies were found. In addition, the KO mice demonstrated circulating autoantibodies to joint tissue, but not to the kidney or spleen. Employing an autoantigen array, antibodies to joint constituents were identified in the KO mice, although these may be different from those identified using the immunoblot analysis. Of particular interest is aggrecan, which when immunized into BALB/c mice results in autoreactive T cells and destructive arthritis^31^, while patients with RA exhibit aggrecan-reactive T cells^32^. Although collagen VI is found in a variety of tissues, it is a major component of the chondrocyte pericelluar matrix, and deletion results in osteoarthritis^33^. Autoantibodies to α-actinin have been described in lupus glomerulonephritis^34^ and autoimmune hepatitis^35^; however, no autoimmunity to the kidney or liver was detected in the CD11c-Flip-KO mice.

Autoreactive CD4^+^ T cells were identified in the CD11c-Flip-KO mice. DCs may restrain autoimmunity centrally through negative selection and the generation of natural Tregs in the thymus or by promoting CD4^+^ T-cell anergy or the generation of inducible Tregs from recent thymic emigrants in the periphery^2^. Both CD8α^+^ and CD8α^−^ DCs are capable of processing foreign or endogenous antigen and presenting them to CD4^+^ T cells, while CD8α^+^ DCs are specialized to cross-present these antigens to CD8^+^ T cells, critical for the development of protective cytotoxic T-cell responses^36^. Cross-presentation by DCs may also contribute to thymic-negative selection^4^,^5^,^6^,^37^. Nonetheless, despite the reduction of CD8α^+^ DCs in the thymus, no defects in central tolerance were identified in the CD11c-Flip-KO mice.

Recently, emigrated CD4^+^CD8^−^Foxp3^−^ thymocytes are the preferential precursors for differentiation of inducible Foxp3^+^ Tregs peripherally^38^. Furthermore, splenic CD8α^+^ cDCs are critical for the induction of Tregs in the periphery^10^, suggesting that the generation of inducible Tregs may also be deficient in CD11c-Flip-KO mice. However, Langerin^+^ migratory DCs were more potent than lymphoid-resident DCs in generating antigen-specific, inducible Tregs^39^, which were not specifically examined in this manuscript. Further studies will be required to fully define the role of CD8α^+^ cDCs in the reduction of Tregs observed in the CD11c-Flip-KO mice.

There was a modest lymphopenia in the CD11c-Flip-KO mice, which was most likely because of the small size of the thymus. No defects in thymocyte development or positive or negative selection were identified. Reduced thymic size in the KO mice was, however, associated with decreased ETPs. The mechanism for the diminished ETPs is unclear, since the bone marrow progenitors, especially the LSK cells, were actually increased, although there was a limitation in the progression from LSK to LMPP cells in the bone marrow of the KO mice. Since differentiation into LMPPs is accompanied by upregulation of CCR7 and CCR9, which are necessary for thymic settling, it is possible that reduction of CCR7 or 9, or P-selectin or integrins^40^,^41^, may contribute to the reduced number of ETPs observed in the CD11c-Flip-KO mice. Nonetheless, lymphopenia is a common feature of many models of RA, including K/BxN, SKG and TS1xHACII mice^42^,^43^,^44^,^45^, and it is also seen in patients with RA^46^,^47^. Lymphopenia may be followed by homeostatic proliferation, which may include autoreactive T cells that escape negative selection, contributing to autoreactivity and arthritis^45^. The transfer of syngenic T cells into K/BxN mice diminished pathogenic T cells, CD4^+^ T-cell proliferation and prevented the development of arthritis^45^. Supporting a role for lymphopenia, in the CD11c-Flip-KO mice increased CD4^+^ and CD8^+^ memory T cells, are consistent with homeostatic proliferation^21^, were identified in the pLN-draining inflamed joints; however, only the CD4^+^ subset was autoreactive.

The reduction of Tregs was at least permissive for the development of autoreactive T cells. Even though there was a significant reduction of Tregs, and the number in the spleen inversely correlated with arthritis, the cause of the arthritis is more complex since deletion of Tregs results in fatal autoimmunity, with autoreactive T cells infiltrates in the pancreas, thyroid and intestine^11^,^48^, which were not observed in the CD11c-Flip-KO mice. However, Tregs may also be important contributors to inflammatory arthritis and joint destruction. CD4^+^CD25^+^ Tregs suppress collagen-induced arthritis and inhibit bone destruction that results from inflammatory arthritis^49^,^50^. Consistent with our observations, in the K/BxN model, in the presence of lymphopenia, Tregs were critical in suppressing pathogenic, autoreactive T cells 44 and the expression of pathogenic autoantibodies^51^. In addition, it is possible that the reduction of NK cells identified in the CD11c-Flip-KO mice may have contributed to the autoimmune phenotype. Supporting this possibility, CpG oligonucleotides mediated cross-talk between CD8α^+^ DCs and NK cells, which induced suppression of K/BxN serum transfer-induced arthritis^52^. Furthermore, since red pulp macrophages were reduced in the spleen of CD11c-Flip-KO mice at 4 weeks, their potential role in the development of systemic autoimmunity cannot be excluded. However, to date no study has identified a direct role for these cells in the pathogenesis of RA.

A number of observations suggest that the CD11c-Flip-KO mouse is a novel model that may provide important insights into the pathogenesis of RA. As observed in patients with RA, the KO mice are lymphopenic. Furthermore, patients with RA exhibit reduced Treg activity and the number of Tregs may be reduced, inversely correlating with disease activity^53^,^54^. The CD11c-Flip-KO mice exhibit a reduction of thymocytes available for emigration to the periphery, while the bone marrow precursors were elevated and patients with RA demonstrate a deficiency of recent thymic emigrants despite an increase in the pre-thymic precursors^55^. In addition, *CFLAR* (*FLIP*) has recently been identified as a risk locus in RA^14^. This same study identified the greatest enrichment of epigenetic modifications of RA risk alleles in Tregs, further supporting a critical role of Tregs in the pathogenesis of RA. Further, similar to the CD11c-Flip-KO mice, Flt3L is increased in patients with RA^56^, suggesting a relative reduction of DCs in RA. The paucity of RA-specific anti-CCP antibodies in the CD11c-Flip-KO mice may be due to the absence of the appropriate genetic background. Future studies crossing the CD11c-Flip-KO mice with mice transgenic for RA shared epitope HLA-DRB1*0401 will be of interest to determine whether the repertoire of autoantibodies is changed to more closely resemble those identified in seropositive RA.

## Methods

### Mouse lines

*Flip*^*flox/flox*^ mice, generated on a C57Bl/6 background, and described previously^19^, were crossed with CD11c-Cre-GFP transgenic mice (Jackson Laboratory)^17^, expressing bicistronic GFP and Cre recombinase driven by a CD11c promoter. Mice genotyped as *Flip*^*flox/flox*^, *CD11c*^*cre*^ have *Flip* deleted in CD11c^+^ cells (CD11c-Flip-KO). To avoid confusion with lines that might be derived from other breeders, this line may more specifically be referred to as the HUPO mouse. Littermates with *Flip*^*flox/+*^, *CD11c*^*cre*^ or *Flip*^*flox/flox*^, *CD11c*^*+*^ served as controls. Both male and female mice were used for experiments. Unless otherwise indicated, the mice were grouped by age as 4 weeks (range 3.5∼4.5 weeks) or ≥20 weeks (range from 20- to 26-week-old) for the CD11c-Flip-KO mice or the littermate controls, CD45.1, *Rag*^*−/−*^, RIP-mOVA and OTII TCR-transgenic mice are from Jackson Laboratory. *Rag*^*−/−*^ mice were crossed with CD11c-Flip-KO mice to generate the *Rag*^*−/−*^-*Flip*^*flox,flox*^*, CD11*^*cre*^ (KO-*Rag*^*−/−*^) mice. Both genders were used for these experiments. The OTII line was crossed with CD45.1 mice to generate the OTII, CD45.1 line and with CD11c-Flip-KO mice to generate the OTII-*Flip*^*flox/flox*^, *CD11c*^*+*^(OTII-control) and OTII-*Flip*^*flox/flox*^, *CD11c*^*cre*^ (OTII-CD11c-Flip-KO) lines. Only males are OTII+ and were therefore used in breeding and the experiments. Bone marrow chimeras were generated employing male RIP-mOVA as hosts, which were lethally irradiated with 1,100 rads followed by retro-orbital administration of 5 × 10^6^ bone marrow cells from male OTII-control or OTII-CD11c-Flip-KO mice. All mice were breed on the C57Bl/6 background. All genotyping was performed by PCR employing genomic DNA extracted from tail biopsies. All animal procedures followed the local ethical guidelines and were approved by the Office of Research Safety and the Institutional Animal Care and Use Committee of Northwestern University.

### Evaluation of arthritis

CD11c-Flip-KO and the littermates were assessed for the spontaneous development of arthritis on a weekly basis from 4 to 36 weeks of age. The clinical severity score was defined as the sum (maximum=28) of joint swelling/inflammation (0–3 each of four paws/ankles), joint deformity (including toe flexion, contraction and shortening, 0–3 of each of four paws/ankles) and the grip strength (on a 3-mm diameter of wires, 0–4 per mouse)^57^. The clinical incidence was defined as any mouse with clinically observed joint swelling/inflammation ≥1. The histologic analysis was performed utilizing paraffin-embedded sections of interphalangeal joints of the back and front paws, ankles and knees subjected to haematoxylin and eosin (H&E) staining^58^. H&E ankle sections were evaluated by a blinded pathologist for inflammation (0–5), bone erosion (0–5), pannus formation (0–5) and median synovial lining thickness, neutrophil infiltration and cartilage destruction^58^,^59^. Macrophages in ankles were identified with immunohistochemistry staining by the Northwestern University Mouse Histology & Phenotyping Laboratory employing anti-F4/80 (eBioscience, cat. no. 14–4801, 1:1,000) or isotype-matched control IgG (eBioscience, cat. no. 16–4321) antibodies.

### Immunoblotting

Tissue homogenates from the ankles, kidney, spleen and liver were harvested from *Rag*^*−/−*^ mice immediately after perfusion with 10–15 ml of PBS. To prepare the ankles, they were de-skinned, dissected and the open ends flushed with PBS using 25G needle to remove the bone marrow. Immunblots were performed as described^60^. Specifically, the tissues were homogenized in cell lysis buffer, supplemented with 1 × protease inhibitor (Sigma-Aldrich). The total protein extracts (100 μg) were resolved with 12% SDS–PAGE and transferred to polyvinylidene difluoride membranes (Immobilon-P; Millipore). The membranes were then incubated with serum (1:100) from individual mice overnight at 4 °C, followed by horseradish peroxidase-linked (HRP) anti-mouse IgG secondary antibody (GE Healthcare, 1:5,000) for 2 h at room temperature. The immunoblots were developed using an ECL Prime Western blotting detection reagent (GE Healthcare) and the UltraLum image acquisition system. A duplicate tissue blot was stained with Coomassie blue R250.

### Cell preparation and immunophenotyping

Cells were isolated from the thymus, spleen and MxLNs, which included cervical, branchial, axillary, ingunal LNs or the pLNs draining the ankles and hind paws, for the indicated experiments. Cells were also eluted from dissected knee and ankle joints^59^. Single-cell solutions from each of these tissues were prepared following collagenase (1 mg ml^−1^) and DNase I (0.1 mg ml^−1^) digestion. Cell types were determined with immunophenotyping, employing multicolour fluorochrome-conjugated antibodies. The antibodies used to identify cell surface markers were purchased from BD Biosciences (B220; RA3–6B2, CD3; 17A2, CD11b; M1/70, CD11c; HL3, CD19; 1D3, CD25; PC61, CD44; IM7, CD45.1; A20, CD45.2; 104, CD62L; MEL-14, CD64; X54–517.1, CD138; 281-2, Ly6G; 1A8, NK-1.1; PK136, Vα2 TCR; B20.1), eBioscience (CD4; RM4–5, CD8; 53–6.7, F4/80; BM8, MHCII; M5/114.15.2) or Miltenyi Biotec (mPDCA-1; JF05-1C2.4.1, 1:10 dilution) and used at a dilution of 1:100–1:500, unless otherwise noted. After surface-staining, Foxp3 (eBioscience, FJK-16s), interferon-γ (eBioscience, XMG1.2) IL-17 (eBioscience, eBio17B7) and TCR-β (BD Bioscience, H57–597) were assessed with intracellular staining using fixation/permeabilization reagents purchased from eBioscience (cat. no. 00-5521-00)^58^. Activated caspase-8 was determined employing an antibody to cleaved caspase-8 (Cell Signaling, D5B2, 1:100), utilizing the manufacturer’s protocol. Data were acquired employing BD LSR II flow cytometer and analysed with FlowJo (TreeStar Inc.).

### ELISA

Inflammatory cytokines, chemokines and growth factors in ankles or sera were determined using ELISAs specific for each molecule (DuoSets, R&D). Ankles were homogenized in PBS supplemented with a protease inhibitor cocktail (Sigma). Supernatants were collected using centrifugation at 12,000*g* for 5 min at 4 °C, and the protein concentration was determined using bicinchoninic acid protein assay reagents (Thermo Scientific)^58^,^60^. Immunoglobulins were quantified using ELISA (Southern Biotechnology Association). Antinuclear antibodies (ANA) were determined utilizing a mouse ANA ELISA kit (Alpha Diagnostic International). RF in serum diluted 1:25 was detected employing 1 μg ml^−1^ rabbit IgG (Dako, X0903)-coated ELISA plates^61^. ELISA for antiCCP (anti-CCP) antibodies was performed as described with modification^62^. Briefly, biotin-conjugated CCP peptides were synthesized (ThermoFisher) and bounded (1 μg ml^−1^) to NeutrAvidin-coated microplates (ThermoFisher). Mouse sera (1:100) were incubated for 1 h at room temperature followed by incubation with HRP-conjugated sheep anti-mouse IgG. The results are presented as the product of OD_450 nm_ × serum dilution. Previously described methods were employed to generate synovial proteome microarrays, which were probed with mouse serum^63^.

### *In vitro* cell culture

Unless otherwise stated, *in vitro* cell culture was performed employing RPMI 1640 medium supplemented with 10% (v/v) FBS, 100 U penicillin-streptomycin and 0.1% (v/v) of 2-Mercaptoethanol, at 37 °C in 5% CO_2_ incubator. For intracellular cytokine production by T cells, cells directly obtained from the spleen and MxLNs were cultured in the medium with or without 50 ng ml^−1^ phorbol myristate acetate, 750 ng ml^−1^ ionomycin and 10 μg ml^−1^ brefeldin A (all from Sigma-Aldrich) for 16 h.

### DC survival and differentiation

DC apoptosis was assessed with Annexin V and cell survival by the exclusion of 7-amino-actinomycin (7AAD, BD Bioscience, cat. No. 559763), examined using flow cytometry. Apoptotic cells were identified as Annexin V^+^ and 7AAD^−^, and necrotic cells as 7AAD^+^. To examine DC survival during differentiation, Lin^−^ haematopoietic stem cells from the bone marrow of *Flip*^*f/+*^ or *Flip*^*f/f*^ mice were isolated employing the mouse haematopoietic progenitor cell enrichment kit (StemCell Technologies) according to the manufacturer’s instructions. These freshly isolated Lin^−^ cells were cultured in the StemSpan (StemCell Technologies) medium supplemented with stem cell factor (50 ng ml^−1^), IL-3 (10 ng ml^−1^) and IL-6 (10 ng ml^−1^) overnight, followed by infection with recombinant retroviral vectors expressing GFP alone or GFP plus Cre^19^. The cells were then differentiated in Flt3L (100 ng ml^−1^, Shenandoah Biotechnology) plus GM-CSF (300 pg ml^−1^, R&D Systems) for up to 6 days. Flow cytometry was employed to identify infected cells (GFP^+^) and DC differentiation (CD11c^+^). In order to identify whether apoptosis or RIP1 contributed to the lack of DC differentiation with the cells from the *Flip*^*f/f*^ mice, differentiation was induced in media supplemented with the caspase-8 inhibitor IETD-fmk (40 μM, MP Biochemical), the pan caspase inhibitor zVAD-fmk (10 μM, MP Biochemical), or the RIP1 inhibitor Necrostatin-1 (30 μM, Nec-, Enzo Life Sciences). The concentrations of these inhibitors were optimized using mouse peripheral monocytes.

### Syngeneic mixed lymphocyte response

For the syngeneic mixed lymphocyte response *in vitro*, CD45.2^+^ T cells from pLNs and brachial LNs were isolated and CFSE (Life Technologies, cat. no. C34554, 5 μM)-labelled. Spleen cells from syngeneic CD45.1^+^ mice were depleted of T cells using CD90.2 selection (StemCell Technologies), and used as antigen-presenting cells. Cells were co-cultured for 3, or 5–7 days, harvested and assessed for T-cell proliferation by CFSE dilution, gating on CD45.2^+^CD3^+^CD4^+^ cells. To evaluate for the presence of autoreactive T cells *in vivo,* CFSE-labelled T cells (10–20 × 10^6^ 200 μl^−1^ PBS) from the spleens and LNs were adoptively transferred into syngeneic CD45.1 mice via retro-orbital injections. Eight to eleven days after transfer, spleens and MxLNs from the recipients were examined for intensity of CFSE in CD45.2^+^ T cells.

### Treg function

Treg function was examined using CD4^+^CD25^+^ cells isolated from the spleens of control or KO mice and CD4^+^CD25^−^ T responder (Tresp) cells were from spleens of CD45.1 mice, utilizing the Regulatory T Cell Isolation Kit from Miltenyi Biotech. Serial dilutions of Treg (starting at 1 × 10^5^) were co-cultured with CFSE-labelled Tresp cells (0.5 × 10^5^), incubated in anti-CD3 monoclonal antibody (mAb; BD Bioscience, cat. no. 553057, 1 μg ml^−1^)-coated round-bottomed plates in the presence of irradiated splenocytes (1 × 10^5^) for 3 days. Proliferation of Tresp cells was determined using CFSE dilution, gating on CD45.1^+^ cells. In addition, the ability of Tregs to suppress autoreactive T cells was examined employing the syngeneic mixed lymphocyte response as described above. For this assay, the Tregs were labelled with 1 μM of PKH26 red fluorescent cell liker, following the manufacturer’s instructions (Sigma-Aldrich). Tresp cells were CFSE-labelled CD45.2^+^ T cells from pLNs and brachial LNs of CD11c-Flip-KO mice. The Tregs were isolated from CD11c-Flip-KO or littermate control mice. The 3 × 10^5^ Treg and Tresp cells were co-cultured for 5–6 days. Proliferation of Tresp cells was determined with CFSE dilution, gating on CD45.2^+^ cells. *In vivo* Treg function was performed by adoptive transfer of CD4^+^CD25^+^ cells isolated from the spleens of normal CD45.1 mice into CD11c-Flip-KO mice with arthritis.

### *In vitro* assays for antigen presentation by cDCs

In order to generate bone marrow-differentiated dendritic cells (BMDDCs) for antigen presentation, nucleated bone marrow cells were isolated from the femurs and tibias followed by culture in the presence of recombinant mouse Flt3L (100 ng ml^−1^) plus GM-CSF (300 pg ml^−1^) for 8 days^64^. In addition, CD11c^+^ cells were isolated from spleens by positive selection (Miltenyi Biotech). CD4^+^ T cells were isolated by negative selection (StemCell Technologies) from the spleens of OTII/CD45.1 mice. Antigen presentation was performed by co-culture of purified splenic DCs (0.5 × 10^4^) or BMDDC with CFSE-labelled CD4^+^ OTII (1 × 10^5^) in the absence or presence of OVA_323–339_ peptide (EZ Biolab, 0.1 μg ml^−1^), in round-bottomed 96-well plates for ∼65 h. Cells were harvested and assessed for proliferation of OTII+ T cells by dilution of CFSE in CD45.1^+^CD4^+^*Va2*^+^ cells^65^.

### *In vitro* assays for T-cell activation and proliferation

For anti-CD3/CD28-induced T-cell proliferation, total T cells were isolated by negative selection (StemCell Technologies). T cells from the spleen or MxLNs were labelled by CFSE and cultured for 3 days in plates coated with anti-CD3 (1 μg ml^−1^) in the presence of anti-CD28 mAb (BD Bioscience, cat. no. 553295, 1 μg ml^−1^). Proliferation was determined with CFSE dilution.

### T-cell adoptive transfer for homeostatic proliferation

T-cell homeostatic proliferation employed CFSE-labelled T cells (10–20 × 10^6^ 200 μl^−1^ PBS), supplemented with B cells (5 × 10^6^) from syngeneic CD45.1 mice, adoptively transferred into *Rag*^*−/−*^ mice. Three to six days after the transfer, the spleen and MxLNs from the recipients were examined.

### Statistical analysis

All quantitative data are presented as mean±s.e.m. Statistical analysis between the two groups was performed with unpaired or paired two-tailed Student’s *t*-test. For samples that failed the normality test, Mann–Whitney rank test was performed. Correlations were determined by Pearson’s linear correlation. For multiple comparisons, one-way ANOVA followed by Tukey’s pairwise mean comparison for multigroup analysis, or, if the samples failed the normality test, with the Dunn’s test of multiple comparisons, was implemented. The Bonferroni correction was performed for the analysis of the autoantigen antibody array by multiplying the *P* value for the difference between the results of the CD11c-Flip-KO and control mice by the number of autoantigens tested (*p* × 123), and reported as a corrected *P* value (*p*_c_). Significance levels were set at 0.05.

## Additional information

**How to cite this article:** Huang, Q.-Q. *et al.* CD11c-mediated deletion of Flip promotes autoreactivity and inflammatory arthritis. *Nat. Commun.* 6:7086 doi: 10.1038/ncomms8086 (2015).

## Supplementary Material

Supplementary Information Supplementary Figures 1-7

## Figures and Tables

**Table 1:** T and B lymphocytes in peripheral lymphoid organs.

		**Spleen**				**Mixed LNs (10 nodes)**			
		**4 Weeks**		**≥20 Weeks**		**4 Weeks**		**≥20 Weeks**	
		**%**	**No. ( × 10**^**6**^)	**%**	**No. ( × 10**^**6**^)	**%**	**No. ( × 10**^**6**^)	**%**	**No. ( × 10**^**6**^)
**CD4^+^T**	Control	10.7±0.5	10.4±1.0	14.2±0.4	17.1±1.3	28.5±1.5	12.6±0.9	18.5±0.8	9.8±1.1
	KO	6.6±0.4 ↓ *** *	6.0±0.8 ↓ ***	11.7±0.4 ↓ ***	16.8±0.9	16.0±0.8 ↓ ***	8.5±0.9 ↓ **	17.0±0.9	16.8±1.7 ↑ **
									
**CD8^+^T**	Control	4.3±0.3	4.1±0.4	8.3±0.4	9.9±0.8	12.9±0.5	5.6±0.5	15.8±1.1	8.4±1.0
	KO	2.0±0.2 ↓ ***	2.0±0.3 ↓ ***	4.0±0.3 ↓ ***	5.6±0.5 ↓ ***	9.9±0.4 ↓ ***	5.2±0.5	11.7±1.0 ↓ **	11.3±1.2
									
**B-cell**	Control	38.2±1.4	39.6±4.4	28.1±1.3	24.3±2.6	26.9±2.2	9.8±1.5	20.4±1.5	11.0±1.6
	KO	17.5±1.6 ↓ ***	15.6±2.2 ↓ ***	33.9±1.6 ↑ **	45.0±4.2 ↑ ***	34.0±1.5 ↑ *	17.4±1.6 ↑ *	31.6±2.3 ↑ ***	43.5±7.5 ↑ ***
									
**CD4^+^Th1**	Control	9.0±1.3		27.4±4.3		2.7±0.3		11.1±2.0	
	KO	7.7±0.9		11.8±3.7 ↓ *		5.7±1.2 ↑*		5.1±0.7 ↓ *	
									
**CD4^+^Th17**	Control	0.7±0.2		0.8±0.2		0.8±0.2		2.4±0.2	
	KO	0.8±0.1		2.9±0.6 ↑*		0.6±0.2		4.3±0.6	

Abbreviations: DC, dendritic cell; KO, CD11c-Flip-knockout; IFN, interferon; IL, interleukin; PMA, phorbol myristate acetate.

Values in bold indicate a significant increase (**↑**) or decrease (**↓**) compared with the littermate control group. *P<0.05, **P<0.01 and ***P<0.001 (unpaired two-tailed *t*-test).†For Th1 and TH17, on PMA/ionomycin activation of CD4^+^CD3^+^ population. *n*=12–27 for CD4^+^ and CD8^+^ T cells; 7–15 for B cells; 3–11 for Th1 and Th17 cells.
