# Supplementary material for: CD11c-mediated deletion of Flip promotes autoreactivity and inflammatory arthritis
Source: Nat Commun. 2015 May 12;6:7086. doi: 10.1038/ncomms8086 (PMC4429912; doi:10.1038/ncomms8086)
Supplement: Supplementary Information — Supplementary Figures 1-7 [file ncomms8086-s1.pdf]

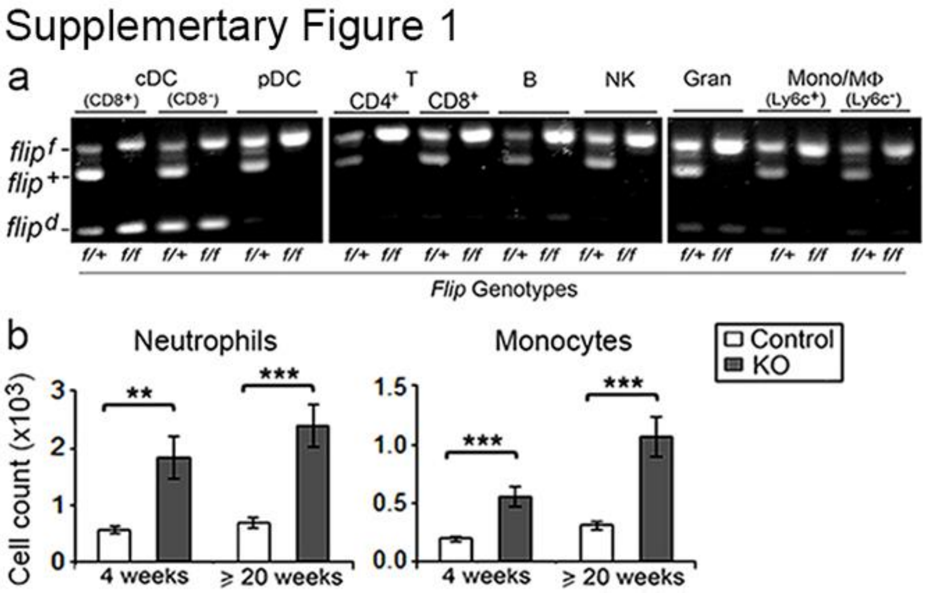

Supplementary Figure 1. Generation of mouse line with conditional deletion of Flip in cDCs results in increased circulating neutrophils and monocytes. (a) PCR genotyping for *Flip*, employed 3 primers: (1) 5'-CTT TTG GCT TTT GGA TCA GTC ATT-3', (2) 5'- GGA ACC ACG AGA AGC CAA CAT-3' and (3) 5'-TCT CTA ACT CAT TCA GCT TCA ACC ACC A-3'. Alleles of *Flip* include the wild-type ($Flip^{+}$, 220bp, primers 1 and 2), the floxed ($Flip^{f}$, 270bp, primers 1 and 2) and the deleted ($Flip^{d}$, 150bp, primers 1 and 3). CD11c directed floxed *Flip* deletion in different cell types was examined employing cells purified from spleens of mice genotyped as $Flip^{flox/+}CD11c^{cre}$ (control) or $Flip^{flox/flox}$ $CD11c^{cre}$ (KO). **(b)** Neutrophils and monocytes were identified from complete blood counts (n = 14-16 for 4 weeks and 8-11 for ≥20 weeks). The data are presented as the mean ±1 SE. (** $p < 0.01$ and *** $p < 0.001$, unpaired 2 sided *t*-test).

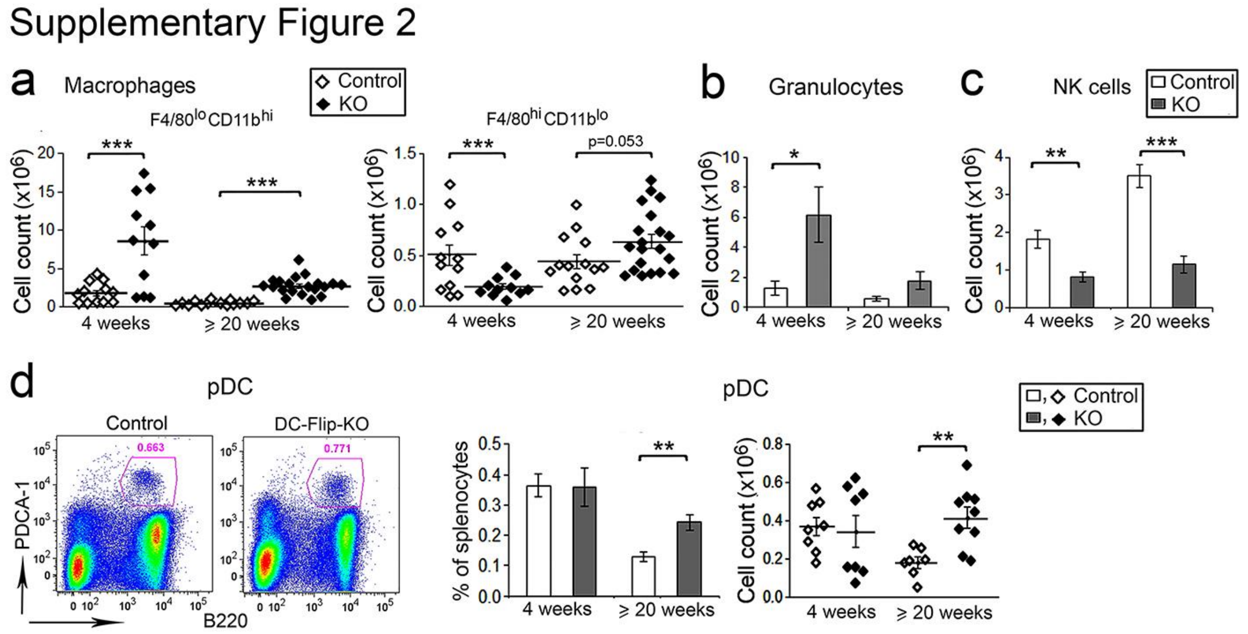

Supplementary Figure 2: Alteration of cells in the spleen of CD11c-Flip-KO mice. (a) Flow cytometry was employed to identify macrophages, defined as $CD64^{+}F4/80^{lo}CD11b^{hi}$ or $CD64^{+}F4/80^{hi}CD11b^{lo}$,**(b)** granulocytes (9-20 per group), defined as $CD64^{-}CD11b^{hi}Ly6G^{+}$, **(c)** NK cells (n = 5 per group), defined as $TCR\alpha\beta^{-}CD11c^{lo}NK1.1^{+}DX5^{+}$ and **(d)** plasmacytoid DCs (pDC), defined as $CD64^{-}PDCA\text{-}1^{+}B220^{+}CD11c^{lo}$, employing CD11c-Flip-KO (KO) or control mice. The data are presented as the mean ±1 SE. (** represents p < 0.01 and *** p < 0.001, unpaired 2-sided *t*-test).

Supplementary Figure 3

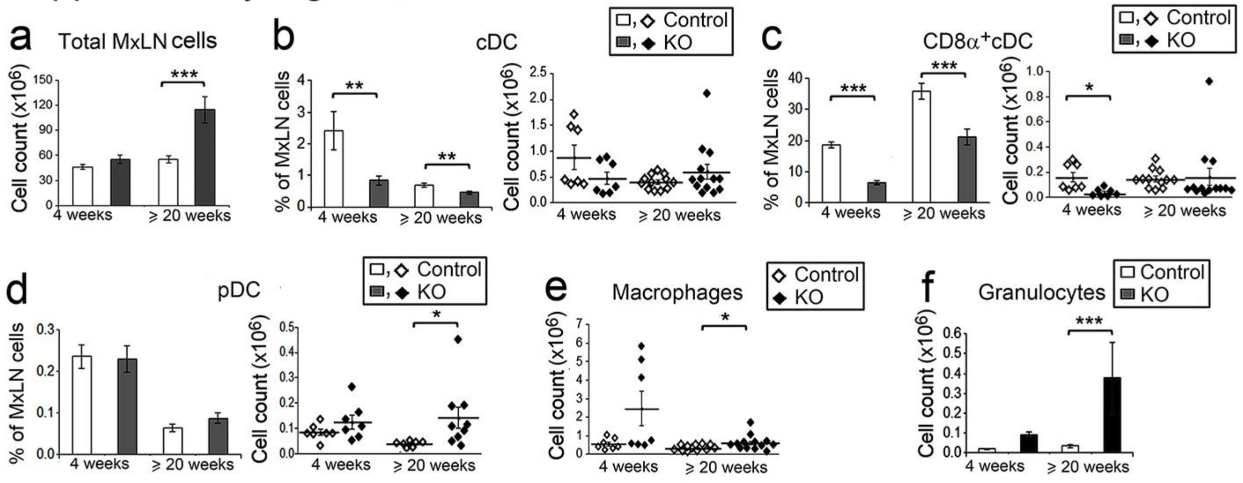

Supplementary Figure 3: Alteration of cells in the MxLN of CD11c-Flip-KO mice. (a) The number of cells in the mixed lymph nodes (MxLNs) (n = 24-29 per group), a mixture of cervical, brachial, axillary, and inguinal LNs, were increased at $\geq$ 20 weeks in the CD11c-Flip-KO (KO) mice. **(b-g)** MxLN cells analyzed by flow cytometry for **(b)** total $CD11c^+$ DC, **(c)** the $CD8\alpha^+$ cDC subset, **(d)** pDC, **(e)** macrophages, defined as $CD64^+F4/80^+CD11b^+$, and **(f)** granulocytes (n = 4 at 4 weeks and 13-16 at $\geq$ 20 weeks). The data are presented as the mean ±1 SE. (* represents $p < 0.05$, ** $p < 0.01$ and *** $p < 0.001$, unpaired 2-sided *t*-test).

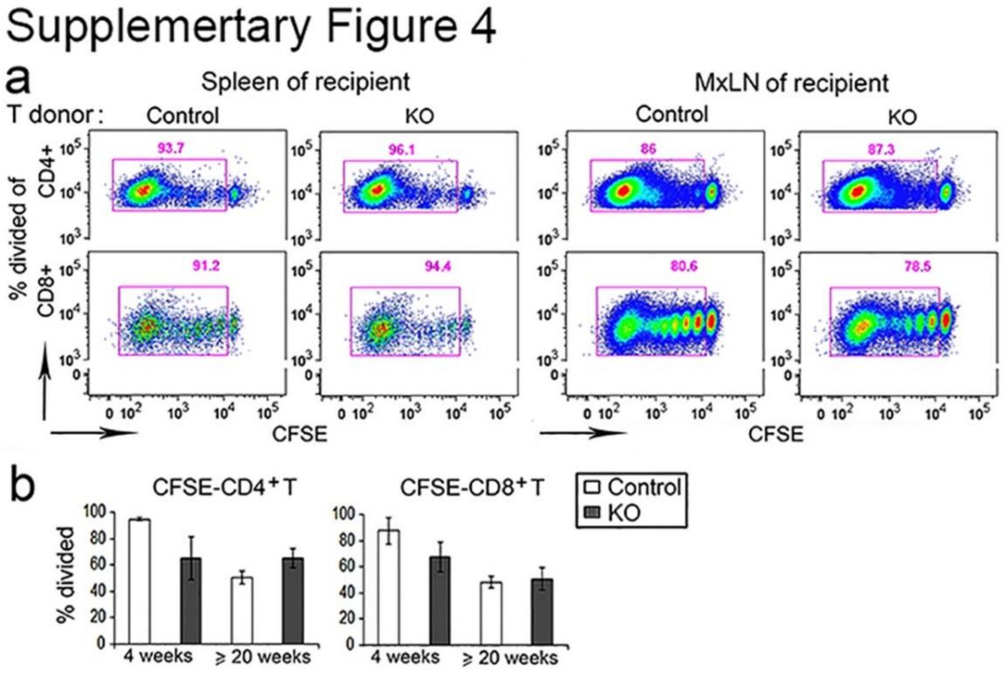

Supplementary Figure 4: Normal CD11c-Flip-KO T cell expansion and proliferation. (a) The homeostatic proliferation of total T cells from DC-Flip-KO (KO) or littermate controls was examined *in vivo* by adoptive transfer into *Rag*$^{-/-}$ mice, employing CFSE-labelled T cells (10-20x10^6 200μl^{-1} PBS), supplemented with B cells (5x10^6). Data shown are representative for spleen (left) and MxLNs (right) from the recipients collected 6 days after transfer. The results are representative of 3 independent experiments. **(b)** *In vitro* anti-CD3/CD28 induced T cell proliferation. CFSE-labelled T cells were from the spleens of DC-Flip-KO or littermate control mice (n= 3-4 per group). They were cultured with plate bound anti-CD3 monoclonal antibody (1μg ml^{-1}) in the presence of anti-CD28 (1μg ml^{-1}) for 3 days. T cell activation was assessed by the percent reduction of CFSE in the $CD4^+$ or $CD8^+$ cell populations, determined by flow cytometry. The data are presented as the mean ±1 SE.

Supplementary Figure 5

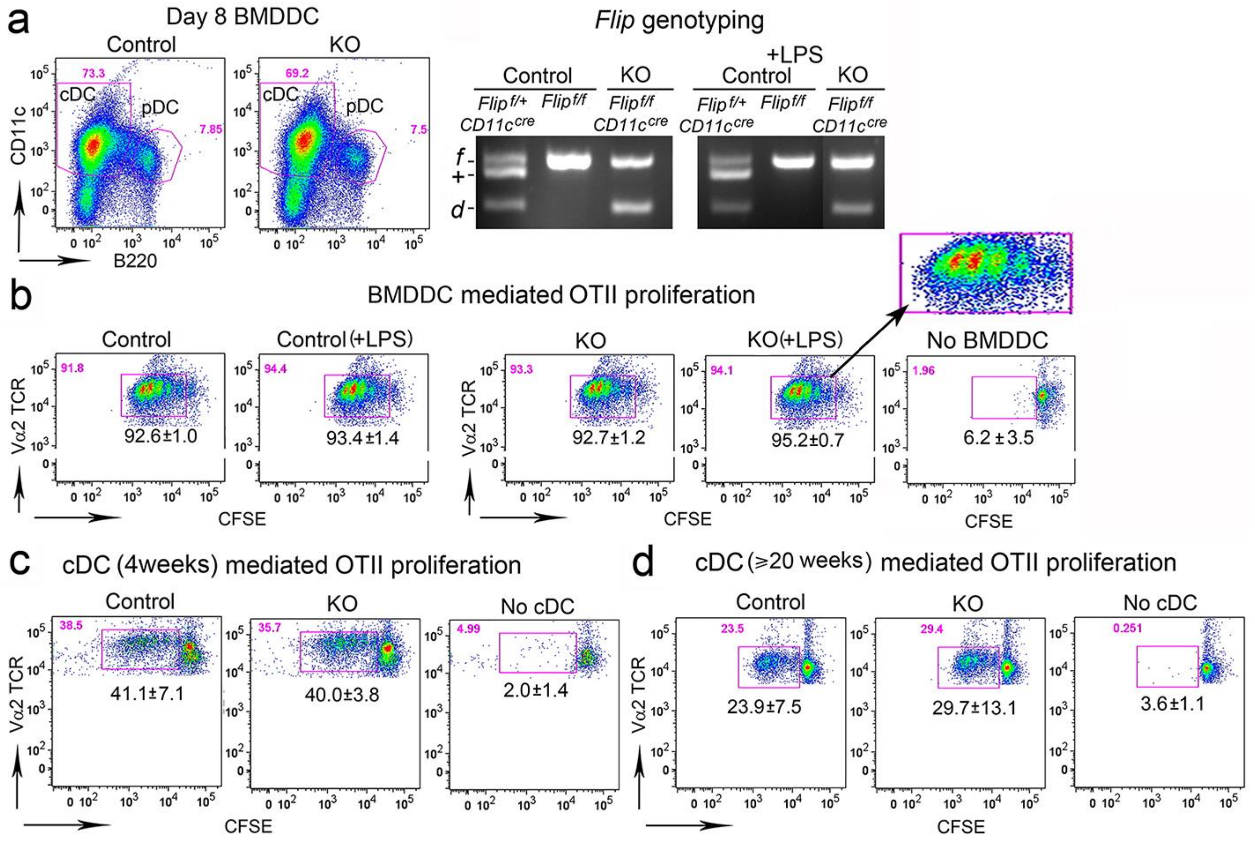

Supplementary Figure 5. No defect of antigen presentation by CD11c from DC-Flip-KO mice. (a) Bone marrow differentiated DCs (BMDDC) from control or CD11c-Flip-KO (KO) mice were gated as CD64$^-$CD11c$^+$B220$^-$ and pDCs are identified as CD64$^-$CD11c^{lo}B220$^+$ (representative of $\geq$ 5) The deletion of *Flip* is confirmed by PCR genotyping (right panel), as described in Supplementary Figure 1. **(b)** BMDDC were examined for their ability to present OVA peptide to CD4$^+$ OTII-T cells, utilizing 0.5x10^4 BMDDC and 1x10^5 CFSE labelled CD4$^+$OTII-T cells. The results are presented as mean ± SE in each representative histogram (n = 3). An example of divided cells in the gated box is enlarged. **(c-d)** CD11c$^+$ DCs were isolated from the spleens of 4 week **(c)** and ≥20 week **(d)** mice and employed as antigen presenting cells. Data presented are from experiments employing 2x10^4 purified DCs and 1x10^5 CFSE-CD4$^+$OTII-T cells. Proliferation of CD4$^+$OTII-T cells was determined by the dilution of CFSE, the % of divided cells in CD4$^+$ Vα2$^+$ population is presented as the mean ± SE for 3-4 mice in each age group.

Supplementary Figure 6

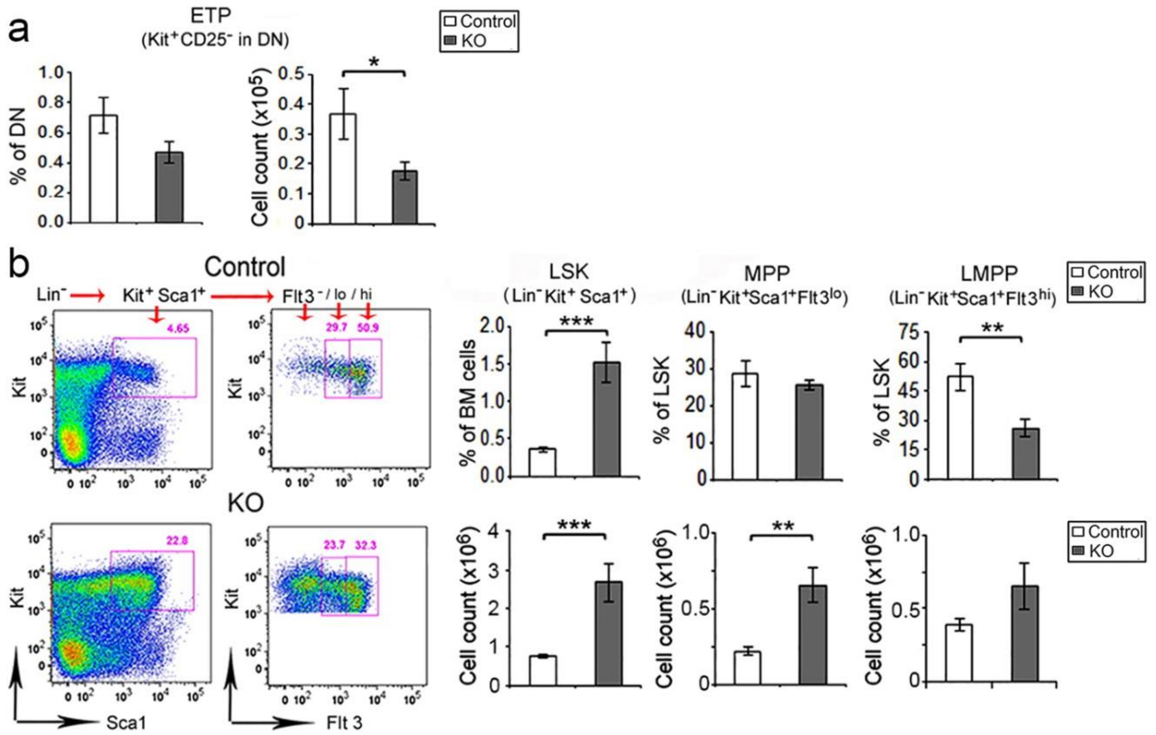

Supplementary Figure 6. Progenitor cells in thymus and bone marrow. (a) Early thymic progenitors (ETP) are defined in $CD4^-CD8^-$ (DN) thymic cells and Kit^+CD25^- (n = 10-11 per group) in the CD11c-Flip-KO (KO) and control mice. **(b)** Hematopoietic progenitors in the bone marrow are analyzed by Flow. The lineage antigens included CD3, CD11b, CD19, B220, Gr-1 and Ter119. The hematopoietic stem cells were identified as $Lin^-Kit^+Sca\ 1^+$ (LSK), and they give rise to downstream multipotent progenitor (MPP, $Lin^-Scal^+Kit^+Flt3^{lo}$), which in turn produce lymphoid-primed multipotent progenitor (LMPP, $Lin^-Scal^+Kit^+Flt3^{hi}$). The DC-Flip-KO and littermate control mice (n = 7 per group) were examined at 4 weeks of age. The data are presented as the mean ±1 SE. (* represents $p < 0.05$, ** $p < 0.01$ and *** $p < 0.001$, unpaired 2-sided *t*-test).

Supplementary Figure 7

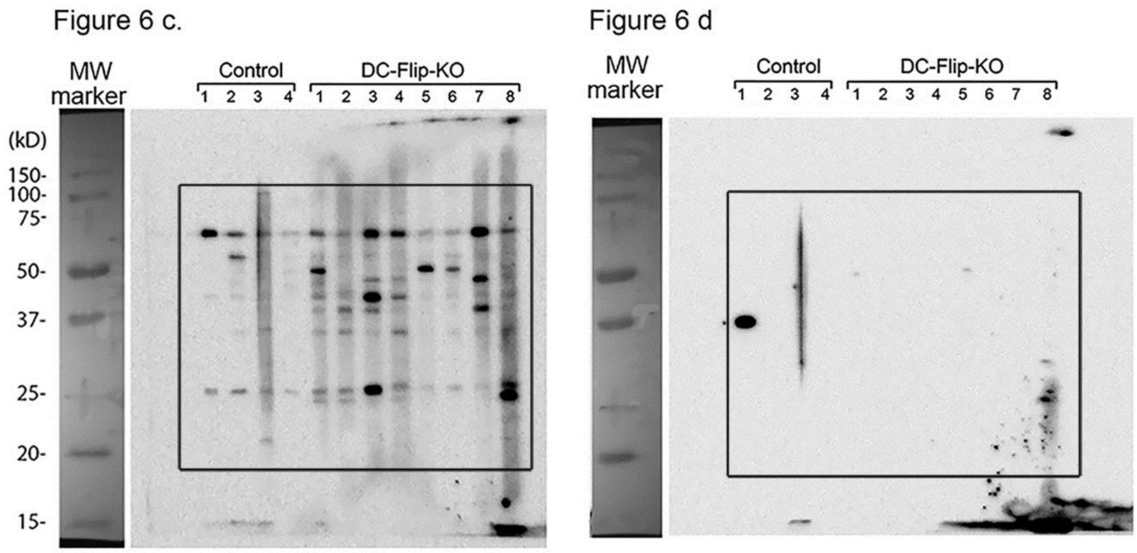

Supplementary Figure 7. The full western blots in Figure 6c and 6d.

The black boxes represent the areas shown in Figure 6. The molecular weight (MW) images were taken from the same gel developed separately.
